# Getting help from the extended family: identification and genetic characterisation of novel resistance to *Globodera pallida* ‘Oberlangen’ in wild *Solanum* species

**DOI:** 10.1007/s11032-025-01582-0

**Published:** 2025-07-22

**Authors:** Helgard Kaufmann, Sebastian Kiewnick, Thilo Hammann, Eckhard Tacke, Stefanie Hartje, Friedrich Kauder, Katja Muders, Vanessa Prigge, Marcus Linde, Thomas Debener

**Affiliations:** 1 Institute of Plant Genetics, Section Molecular Plant Breeding, Leibniz University Hannover, Hannover, Germany; 2 Julius Kühn Institut (JKI) - Institute for Plant Protection in Field Crops and Grassland, Braunschweig, Germany; 3 Julius Kühn-Institut (JKI) - Institute for Breeding Research on Agricultural Crops, Sanitz, Germany; 4 EUROPLANT Innovation GmbH & Co. KG, Lüneburg, Germany; 5 Solana Research GmbH, Windeby, Germany; 6 NORIKA-Nordring-Kartoffelzucht- und Vermehrungs-GmbH, Sanitz, Germany; 7 SaKa Pflanzenzucht GmbH & Co. KG, Windeby, Germany

**Keywords:** Quantitative resistance loci, Sustainable crop protection, Genetic resources, Genebank, Pyramiding, Germplasm

## Abstract

The potato cyst nematode *Globodera pallida* is a major agricultural pest that is responsible for significant potato yield losses on a global scale. The most effective and sustainable strategy to reduce damage caused by *G. pallida* is to cultivate nematode-resistant potato varieties. These varieties have been developed mainly by introgressing resistance genes from wild potato species. However, intensive reliance on limited and widely utilised sources of resistance against the predominant *G. pallida* pathotypes 2 and 3 (Pa2/3) has led to the emergence of virulent nematode populations that can overcome these defences. To address this issue, we undertook a comprehensive search for novel sources of resistance against this new virulent *G. pallida* type, ‘Emsland’, within wild *Solanum* species and identified several promising candidates. Three resistant accessions were further characterised with resistance loci mapped via a single-nucleotide polymorphism array in biparental progenies. Furthermore, one population was subjected to fine-mapping through genotyping-by-sequencing. A major resistance locus was identified in each of the three studied accessions: in a clone of *Solanum gourlayi* ssp. *pachytrichum*, resistance was mapped to the short arm of chromosome 5, whereas in clones of both *S. hawkesianum* and *S. spegazzinii*, resistance was traced to a locus on the long arm of chromosome 6. This study is the first to identify resistance loci controlling the new virulent *G. pallida* type, ‘Emsland’ (population ‘Oberlangen’). Our findings provide essential data for the introgression of these resistance loci into cultivated potato varieties, offering the potential to increase nematode resistance in potato crops.

**Supplementary Information:**

The online version contains supplementary material available at 10.1007/s11032-025-01582-0.

## Introduction

Potatoes are a staple food worldwide, ranking fourth in importance after maize, wheat, and rice. In 2022, 376 million tons of potato were produced worldwide (FAO 2023). Effective management of pests and diseases in potato cultivation is critical for maintaining economic stability and food security. Using natural and environmentally friendly methods for disease and pest control is important for maintaining the sustainability of potato farming.

Potato cyst nematodes (PCNs) significantly limit potato yields. These highly specialised parasites inhabit the root system and form distinctive cysts filled with eggs and juveniles. The most harmful PCN species are *Globodera rostochiensis* (Wollenweber 1923) and *Globodera pallida* (Stone 1972), also known as golden and pale cyst nematodes, respectively. As PCNs are classified as quarantine pests in most countries, farmers are subject to stringent measures to prevent infestations and control these nematodes upon detection (Orlando and Boa 2023).

For decades, breeding resistant potato varieties has been a cornerstone strategy for combating PCN (Ross and Huijsman 1969). The systematic search for resistance genes was initiated by Ellenby, followed by Huijsman’s demonstration of monogenic dominant inheritance, marking significant milestones in the utilisation of genes from wild species for PCN resistance (Ellenby 1948; Huijsman 1955). The numerous accessions of wild species from Central and South America that have been studied are preserved in genebanks worldwide, including the *Gross Luesewitz potato collection* (GLKS) in Germany and the *Center for Genetic Resources*,* the Netherlands* (CGN) (Dellaert and Hoekstra 1987; Rousselle-Bourgeois and Mugniery 1995).

To date, only a few resistance genes effective against *Globodera pallida*, originating from *Solanum vernei*, *Solanum tuberosum spp. andigena*, and *Solanum spegazzinii*, have been introgressed into potato varieties (Bakker et al. 2006; Dalamu et al. 2012; Spychalla and De Jong 2024). European populations of *G. pallida* predominantly belong to pathotype 2 and 3 (Pa2/3). For several years, the resistance gene GpaV_vrn_ has conferred broad but only partial resistance to Pa2/3 (Rouppe van der Voort et al. 2000). The intensive deployment of this quantitative locus combined with short crop rotations has exerted selection pressure, leading to shifts in virulence and the emergence of *G. pallida* populations that can overcome resistance, as exemplified by the emergence of new virulence types, such as the ‘Oberlangen’ population in the Emsland region of Lower Saxony, Germany, where starch production facilities are located (Mwangi et al. 2019b).

This study aimed to identify new sources of resistance against the virulent *G. pallida* population ‘Oberlangen’ that can overcome resistance. To achieve this goal, accessions of *Solanum* species reported to be resistant to Pa2/3 were evaluated for their response to infestation with ‘Oberlangen‘. Progenies from resistant wild clones were subsequently analysed to characterise the genetic loci involved in resistance.

## Materials and methods

### Plant material

Accessions of *Solanum* species were obtained from the CGN and the GLKS of the German Federal ex situ Genebank (IPK, Germany; Table 1). Seeds were surface sterilised, and plantlets were raised in vitro by the potato breeding company EUROPLANT Innovation, NORIKA or SaKa. Rapid propagation and maintenance were conducted using common tissue culture protocols at the corresponding laboratories. Tubers were produced in peat soil under greenhouse conditions.

**Table 1:** Number of accessions showing high levels of resistance (≤ 3% relative susceptibility) in relation to the total number of clones tested in greenhouse experiments against the *G. pallida* population ‘Oberlangen‘

		Number of clones	
Species	Accession number	resistant^a^	tested
*S. bukasovii*	GLKS 30924	0	7
*S. gourlayi ssp. gourlayi*	GLKS 32133	1	13
*S. gourlayi ssp. gourlayi*	GLKS 32135	0	3
*S. gourlayi ssp. gourlayi*	GLKS 32136	0	5
*S. gourlayi ssp. gourlayi*	GLKS 32137	3	11
*S. gourlayi ssp. gourlayi*	GLKS 32140	1	8
*S. gourlayi ssp. gourlayi*	GLKS 32141	0	10
*S. gourlayi ssp. gourlayi*	GLKS 32143	0	5
*S. gourlayi ssp. gourlayi*	GLKS 32145	0	10
*S. gourlayi ssp. pachytrichum*	GLKS 35354	3	10
*S. gourlayi ssp. pachytrichum*	GLKS 35355	1	8
*S. microdontum*	GLKS 30829	3	10
*S. neorossii*	GLKS 32856	4	10
*S. neorossii*	GLKS 32859	5	10
*S. oplocense*	GLKS 32256	n.a.	23
*S. sparsipilum*	GLKS 30976	0	19
*S. stoloniferum*	GLKS 30519	0	18
*S. stoloniferum*	GLKS 30592	0	10
*S. sucrense*	GLKS 30717	0	10
*S. tuberosum ssp. andigena*	GLKS 33020	0	10
*S. tuberosum ssp. andigena*	GLKS 33135	1	10
*S. vernei*	GLKS 30804	0	10
*S. bukasovii f. multidissectum*	CGN 17733	0	7
*S. gourlayi*	CGN 17965	1	14
*S. hawkesianum*	CGN 17888	5	19
*S. hawkesianum*	CGN 17889	7	10
*S. hannemanii*	CGN 17996	2	10
*S. leptophyes*	CGN 18126	0	14
*S. leptophyes*	CGN 21312	0	8
*S. oplocense*	CGN 18085	1	19
*S. sparsipilum*	CGN 17758	5	40
*S. sparsipilum*	CGN 18094	0	22
*S. sparsipilum*	CGN 18220	0	18
*S. spegazzinii*	CGN 17602	9	9
*S. vernei*	CGN 18114	7	48
Total	35	59	445

^a)^ Resistant clones showing ≤ 3% relative susceptibility in relation to the susceptible control ֹ‘Desireeֹ’ on the basis of cyst counts per plant; n.a. not available owing to poor growth of plants

Segregating biparental populations were produced from susceptible, dihaploid *S. tuberosum* clones of the JKI breeding programme as females and resistant clones from three accessions as males (Table 3). The population 5 A-gop was created by crossing GLKS35354-2 (*S. gourlayi* ssp. *pachytrichum*) with JKI-GL-05.1682.07. The population 14 A-hwk was obtained by crossing CGN17888-16 *(S. hawkesianum)* with JKI-GL-08.1206.02. The population 15 A-spg was obtained by crossing the resistant clone CGN17602-2 *(S. spegazzinii)* with JKI-GL-00.1066.13. A minimum of 250 seeds per population were surface sterilised and germinated, and the plants were propagated and maintained in vitro as described above.

Cv. ‘Desiree’ was used as a susceptible control in all the experiments. Cvs. ‘ֹCardoso’ and ‘Amanda’, which are Pa2/3 resistant but susceptible to *G. pallida* ‘Oberlangen’, served as additional controls.

### PCN population

The virulent *G. pallida* population ‘Oberlangen’ described earlier (Mwangi et al. 2019b) was used in this study. The population originated from a potato field in the Emsland region of Lower Saxony and was maintained on the susceptible cultivar ‘Desiree’ at JKI Braunschweig.

### Resistance phenotyping

#### Screening of GLKS and CGN accessions

Genotype screening was performed in either pot experiments using small tubers or tissue culture (TC)-derived plants according to Mwangi et al. (2019a). The experiments were set up in a growth chamber with day/night temperatures of 20/12°C and a 14-hour light period. The substrate for all the experiments was loess soil (Müller and Rumpenhorst 2000) supplemented with slow-release fertiliser at 1.5 g/kg soil (Osmocote Exact Standard^®^; 15% N; 9% P2O5; 12% K2O and 2% MgO). Inoculation was performed using suspensions of *G. pallida* eggs and second-stage juveniles (E + J2; Mwangi et al. 2019a). The inoculum density was adjusted to 10 E + J2/ml soil for the experiments with tubers and 5 E + J2/ml soil for experiments with TC-derived plants. For each genotype, 5 (tubers) or 6 (TC-derived plants) replicates were used. As a susceptible control, the cultivar ‘Desiree’ was used in all the experiments. In addition, either cv. ‘ֹCardoso’ or cv. ‘Amanda’, which are resistant to virulent Pa2/3 *G. pallida* populations, were included in each experiment.

The experiments were terminated 12 weeks after inoculation, and cysts were extracted from each pot by washing the soil through a 250 μm bucket sieve (Mwangi et al. 2019a). For experiments with TC plants, cysts were extracted via an automated soil sample extractor (www.meku-pollaehne.de; Anonymous 2013). Afterwards, the cysts and plant debris were transferred to filter paper and air-dried at room temperature for 3 days. Cysts were counted via a stereomicroscope.

#### Validation of resistant candidate clones

A selection of the genotypes that displayed resistance in the first screen were retested using TC plants as described above. The experiment was set up in a greenhouse with day/night temperatures set at 20/18°C and 16 h of supplemental light. Each genotype was tested with 12 replicates. Cyst extraction and enumeration were performed as described above.

#### Population screening

TC-derived plants were tested in greenhouse experiments. The experiments were conducted in small plastic containers as described by Mwangi et al. (2019a), with a minimum of six replicates. Cyst extraction and enumeration were performed as described above.

### DNA extraction

DNA was extracted from approximately 30 mg of leaf tissue, which was dried and homogenised via a Tissue Lyser II (Qiagen, Hilden, Germany) via a Nucleospin Plant II kit (Macherey-Nagel, Düren, Germany) according to the manufacturer’s protocol. The DNA concentration was determined with a NanoDrop 2000 spectrophotometer (Thermo Fisher Scientific Inc., Waltham, Massachusetts, USA) at 260 nm, and DNA quality was assessed at 260/230 nm and 260/280 nm. DNA integrity was assessed on 1% agarose gels run at 5 V/cm in comparison with λ-DNA standards.

### SNP genotyping using the GGP potato 25 K SNP array

The Geneseek Genomic Profiler Potato 25 K Chip (Neogen Genomics, Lincoln, Nebraska, USA) was used to genotype the populations 5 A-gop, 14 A-hwk and 15 A-spg, as well as the parental genotypes, for 21,027 single-nucleotide polymorphisms (SNPs). Genotyping was performed by Neogen Genomics with SNP data analysis via Genome Studio, diploid version (Illumina, San Diego, USA). SNP positions were reported in *S. tuberosum* Group Phureja DM1-3 516R44 Genome Annotation v4.3 (The Potato Genome Sequencing Consortium 2011; Sharma et al. 2013).

### GBS analysis

A total of 146 genotypes from population 14 A-hwk were subjected to genotyping-by-sequencing (GBS; ddRAD) analysis. The parental genotypes were included in duplicate. The analysis was performed by LGC Genomics (LGC Genomics, Berlin, Germany). GBS libraries were constructed using the restriction enzymes PstI-ApeKI for digestion and the isolation of fragments with a 225 bp mean insert size. Then, 2 × 150 bp paired-end reads were sequenced on an Illumina NextSeq500/550 v2 and a NovaSeq 6000, with a total of 150 million reads. *S. tuberosum* Group Phureja DM 1–3 516R44 Genome Annotation v6.1 (Pham et al. 2020) was used for alignment with BWA-MEM v0.7.12 (Li 2013) and variant calling with Freebayes v1.0.2–16 (Garrison and Marth 2012).

### Data analysis

#### Screening of GLKS and CGN accessions

The relative susceptibility of the potato genotypes tested was calculated for each clone from the mean number of cysts from all replicates as (mean number of cysts (test candidate)/mean number of cysts (standard susceptible control cultivar) * 100%), where the cv. ֹ‘Desireeֹ’ was used as the standard susceptible control. Clones were considered resistant if their relative susceptibility did not exceed 3%.

#### Marker trait associations

Statistical calculations were conducted using the R software, version 3.6.1 (R Core Team (R 2019)). The normality of the data distribution for each population was assessed separately via the Shapiro–Wilk test, with a significance level of 0.05. In the analysis of the populations, the third quartiles of the cyst counts were calculated instead of the means because they are less susceptible to the influence of outliers. The relative susceptibility in comparison to that of the standard control cv. ‘Desiree’ was determined. A nonparametric Kruskal–Wallis rank test was then used to identify significant SNP effects for PCN resistance with the genotyping results of the SNP array or the GBS analysis. To minimise the number of false positive markers, a false discovery rate (FDR) adjustment (Benjamini and Hochberg 1995) was conducted by calculating the q value (Storey and Tibshirani 2003). A q value of 0.05 was chosen as the significance threshold to indicate that a marker was associated with resistance to *G. pallida* ‘Oberlangen‘. The effect size was calculated as the Pearson`s correlation coefficient (r) as the Z statistic divided by the square root of the sample size (N) ($\:r=Z/\sqrt{N)}$ (Fritz et al. 2012).

## Results

### Resistance to ***G. pallida*** ‘Oberlangen’ in Pa2/3-resistant genebank accessions

Greenhouse experiments demonstrated that among the 445 clones initially evaluated, 59 exhibited resistance against the ‘Oberlangen’ population, which was characterised by a relative susceptibility of 3% or less (Table 1). Among the 35 accessions analysed, CGN 17602 (*S. spegazzinii*) presented the highest proportion of resistant clones, with all nine clones tested displaying resistance. This was followed by CGN 17889, with 7 out of 10 clones exhibiting resistance, and GLKS 32859, with 5 out of 10 clones exhibiting resistance. Conversely, 18 of the accessions did not produce any resistant clones (Table 1).

When 34 genotypes displaying resistance in the first screen were retested for resistance against *G. pallida*, eight clones presented intermediate resistance, characterised by relative susceptibilities ranging between 3% and 10% after 12 weeks of greenhouse cultivation (Table 2). Additionally, 23 clones presented a high level of resistance, with 3% or less relative susceptibility. Notably, CGN 17889-1, CGN 17889-10, GLKS 32856-1 and GLKS 32856-9 achieved complete suppression of *G. pallida* (Table 2). Among the 34 clones assessed, only three showed more than 10% susceptibility to *G. pallida*.

**Table 2:** Validation of putatively resistant potato clones from the first screen. Number of cysts per root on different potato genotypes 12 weeks after inoculation with the *G. pallida* population ‘Oberlangen’

Clone	Number of cysts/per root^a^	Relative susceptibility [%]^b^
Desiree	190.5 ± 15.0	100
Cardoso	36.8 ± 6.7	19.3
GLKS 32859-8	62.5 ± 10.6	32.8
CGN 17996-6	32.4 ± 7.6	17.0
CGN 17758-7	20.7 ± 4.9	10.8
CGN 17758-9	18.6 ± 3.1	9.8
GLKS 32856-10	16.5 ± 2.2	8.7
CGN 17888-13	15.2 ± 3.5	8.0
CGN 17758-12	12.4 ± 2.9	6.5
CGN 17758-14	11.8 ± 3.0	6.2
GLKS 32859-7	9.1 ± 2.0	4.8
CGN 17888-18	7.0 ± 4.1	3.7
CGN 18114-2	6.4 ± 5.5	3.4
CGN 17996-3	4.4 ± 1.4	2.3
GLKS 32859-10	3.7 ± 1.4	1.9
CGN 17996-10	3.0 ± 2.0	1.6
CGN 18114-1	2.9 ± 2.6	1.5
CGN 18114-15	2.8 ± 0.7	1.4
CGN 17758-6	2.4 ± 0.9	1.3
GLKS 32859-6	2.2 ± 0.8	1.2
CGN 18114-7	2.0 ± 1.2	1.0
CGN 17888-17	1.7 ± 0.9	0.9
CGN 17889-3	1.7 ± 1.2	0.9
CGN 17889-2	1.3 ± 0.7	0.7
GLKS 35354-2	0.8 ± 0.2	0.4
CGN 17888-16	0.6 ± 0.2	0.3
CGN 17889-4	0.5 ± 0.4	0.3
CGN 18114-14	0.4 ± 0.3	0.2
CGN 17602-3	0.4 ± 0.3	0.2
CGN 17602-12	0.3 ± 0.1	0.2
CGN 17602-2	0.3 ± 0.2	0.1
CGN 17602-6	0.2 ± 0.1	0.1
CGN 17889-10	0.1 ± 0.1	0.0
CGN 17889-1	0.0	0.0
GLKS 32856-2	0.0	0.0
GLKS 32856-9	0.0	0.0

^a)^ Mean number of cysts per root ± standard error, *n* = 12; ^b)^ relative susceptibility in % in relation to the susceptible control ֹ‘Desireeֹ’

### Evaluation of resistance in segregating populations

On the basis of the resistance screens described above, accessions with a relative susceptibility of less than 0.5% were crossed with various susceptible diploid *S. tuberosum* clones. For further genetic analyses, only those cross combinations that generated offspring with a minimum of 75 individuals, which were available after cultivation and multiplication for phenotypic testing, were considered. This selection process resulted in three populations: 5 A-gop, 14 A-hwk, and 15 A-spg.

These populations were assayed for resistance to the *G. pallida* population ‘Oberlangen’. In each inoculation test, cv. ’Desiree’ served as the positive control along with both parents. In greenhouse trials assessing resistance, up to 56% of plantlets grew poorly or died during the test period, as some progeny displayed a low degree of vitality. The results were available for 33 clones of population 5 A-gop from one trial only, for at least two assessments for 54 individuals of population 14 A-hwk and for 85 individuals of population 15 A-spg (Table 3). The detailed results of the phenotyping experiments are listed in Supplementary Tables S1 to S3.

**Table 3:** Characteristics of the three populations examined in this study

Population	Resistant parent	Susceptible parent	No. of clones tested	No. of clones scored for resistance
5 A-gop	35354-2	JKI GL05.1682.07	75	33
14 A-hwk	17888-16	JKI GL08.1206.02	79	54
15 A-spg	17602-2	JKI GL00.1066.13	85	85

**Fig. 1:**
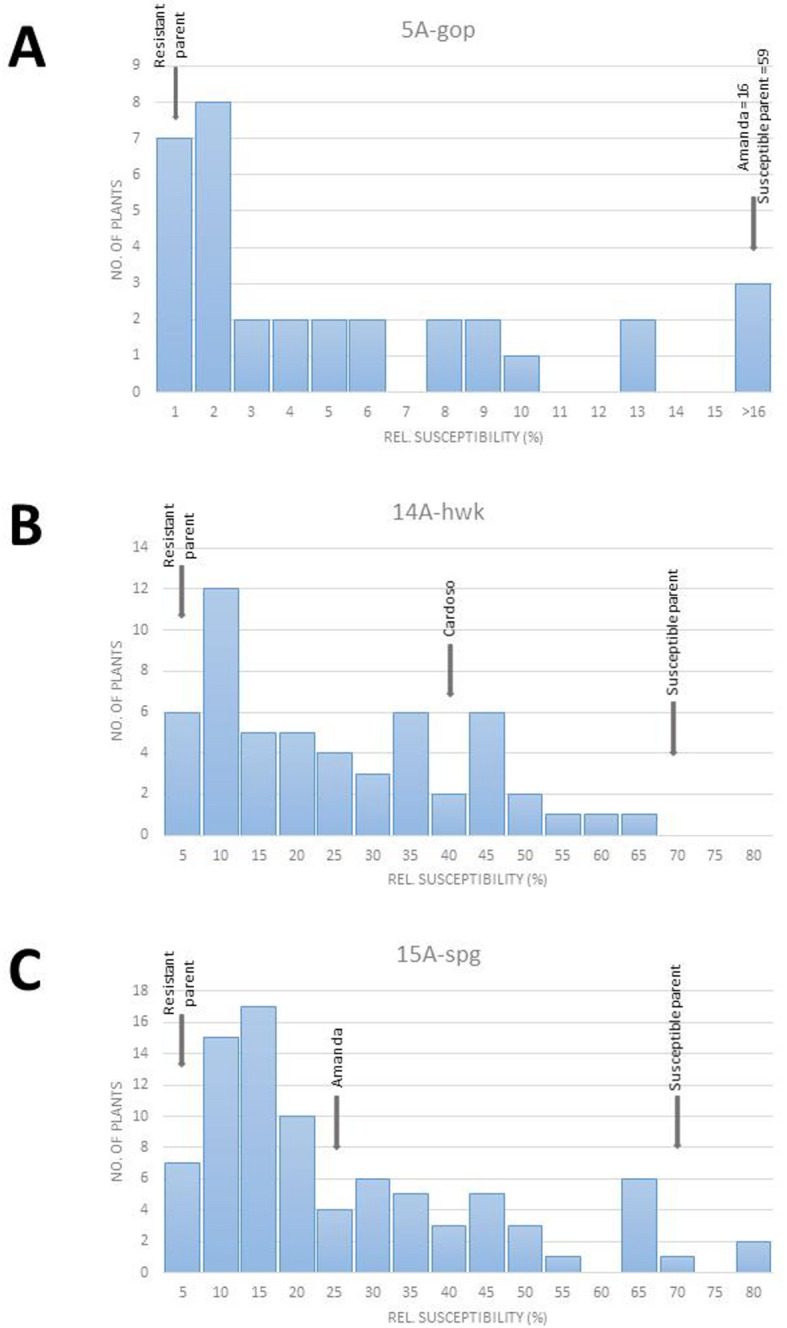
Frequency distribution of the PCN susceptibility of three biparental populations. The average of the third quartiles of the cyst counts from 2 to 3 experiments was calculated relative to the susceptible control ‘Desiree’ in the populations 14 A-hwk (**B**) and 15 A-spg (**C**). For 5Agop (**A**), third quartiles from one experiment were used for the calculation. Susceptibility values for the parental clones and a Pa2/3-resistant cultivar (‘Amanda’ or ‘Cardoso’) are indicated by black arrows

The distribution of the relative susceptibility to *G. pallida* ‘Oberlangen’ in all three populations is shown in Fig. 1. Each population displayed a statistically significant deviation from a normal distribution (Shapiro‒Wilk test, *p* < 0.05). Resistant and susceptible parents were positioned at distribution extremes. The controls included either ‘Amanda’ or ‘Cardoso’, which are resistant to *G. pallida* Pa2/3 but susceptible to ‘Oberlangen’ and whose relative susceptibilities are also depicted. The histogram for population 5 A-gop was extremely right skewed, suggesting the existence of a single dominant resistance locus. In contrast, histograms for populations 14 A-hwk and 15 A-spg indicated possible bimodal or multimodal distributions, suggesting the existence of a major heterozygous resistance locus in the respective resistant parents, possibly influenced by additional minor factors.

### SNP genotyping and genetic mapping

#### Applicability of the GGP SNP array for the analysis of potato species

To map the loci involved in resistance to *G. pallida* ‘Oberlangen’ in three segregating potato populations, SNP data from the GGP 25 K-Potato SNP array were selected for SNPs that were heterozygous in the resistant wild species parents.

Although the SNP call rates were equally high for all samples (0.96–0.98) with a total of > 20,000 calls, the proportion of heterozygous SNPs was much lower in the resistant wild species clones (3.4–5%) than in the susceptible *S. tuberosum* parental clones (20–25%, Table 4). Therefore, the total number of informative SNPs in this study reached a maximum of 1000 in population 5 A-gop, 815 in population 14 A-hwk and only 679 in population 15 A-spg (Table 4). The lowest number of heterozygous SNPs per chromosome was 30 and 33 for chromosome 10 in CGN17888-16 and CGN17602-2, respectively, and 43 for chromosome 6 in GLKS35354-2.

**Table 4:** GGP potato 25 K array genotype calls for the parental genotypes of populations 5 A-gop, 14 A-hwk and 15 A-spg

Clone	Species	No Calls	Calls	Call Rate (%)	A/A Freq (%)	A/B Freq (%)	B/B Freq (%)
V_17602	*S. spegazzinii*	757	20,270	96.40	40.81	3.36	55.83
V_17888	*S. hawkesianum*	726	20,301	96.55	40.20	4.06	55.74
V_35354	*S. gourlayi pach.*	706	20,321	96.64	39.89	4.95	55.16
V_00.1066.13	*S. tuberosum*	394	20,633	98.13	31.92	24.50	43.58
V_08.1206.02	*S. tuberosum*	383	20,644	98.18	33.65	20.86	45.50
V_05.1682.07	*S. tuberosum*	367	20,660	98.25	32.68	22.27	45.05

### Marker trait associations

For genetic mapping, nonparametric Kruskal‒Wallis tests were performed to identify SNPs associated with resistance to *G. pallida* ‘Oberlangen‘. The SNP positions refer to DM1-3 v4.3.

#### Population 5 A-gop

**Fig. 2:**
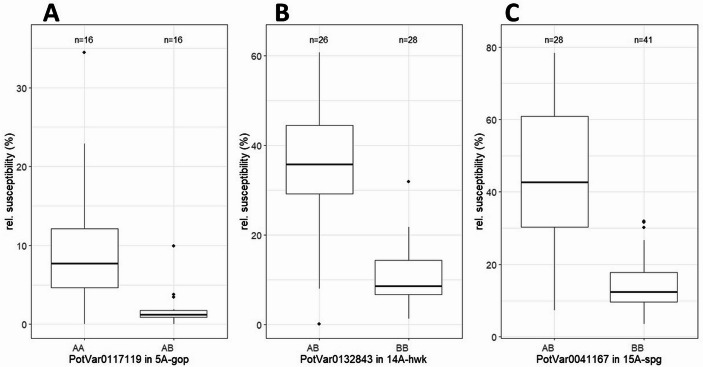
Box plots of relative susceptibility by allele status of a closely linked marker in each of the three segregating populations. The boxes represent the 25th and 75th quartiles, and the medians are indicated by the bold line. **A** Effect of the SNP marker PotVar0117119 on the dependence of the marker alleles in 32 individuals of the 5 A-gop. **B** The SNP marker PotVar0132843 in 54 individuals of 14 A-hwk. **C** The SNP marker PotVar0041167 in 69 individuals of 15 A-spg. For a description of the populations, see the Materials & Methods section

SNP genotyping data were obtained for 32 progeny clones from population 5 A-gop. A total of 1000 SNP markers heterozygous in the resistant parent GLKS35354-2 (*S. gourlayi ssp. pachytrichum)* were analysed for their segregation in the population. Twenty-seven SNPs were significantly associated with resistance to *G. pallida* ‘Oberlangen’, all of which were located within a 6.3 Mb interval on the short arm of chromosome 5 (2,963,857 bp to 9,273,361 bp). The core of this interval ranges from 3,945,412 bp to 5,370,258 bp and is defined by 18 SNPs with the lowest p value of 0.0097 (FDR adjusted). The key features of the SNP marker data are summarised in Table 5. The phenotypic effect of one central SNP marker (PotVar0117119) with a p value of 0.0097 is shown in Fig. 2A. Individuals who were heterozygous for the marker had a median relative susceptibility of 1.2%, whereas homozygotes had a median susceptibility of 7.7%. The calculation of Pearson’s correlation coefficient as a measure of the effect of the marker on relative susceptibility to ‘Oberlangen’ showed a strong effect, with a value of *r* = 0.625. Despite the small sample size, the distance to adjacent markers was relatively short, with values of 0.98 Mb and 0.55 Mb.

**Table 5:** Significant SNP markers associated with *G. pallida* ‘Oberlangen’ resistance in population 5 A-gop. The SNPs delineate a 6.3 mb resistance interval on chromosome 5. Eighteen SNP markers with the lowest p value of 0.009742 are located between 3.94 mb and 5.37 mb in the DM1-3 v4.3 genome assembly. The two markers at the borders of this core interval are presented here in bold type. Table S1 shows the allele configuration of the listed SNPs in population 5 A-gop

SNP	pVal	pValFDR	Position on Chr 05 DM1-3 v4.3	Position on Chr 05 DM1-3 v6.1
PotVar0024727	9.45E-04	0.033771	2,963,857	2,941,826
solcap_snp_c1_3789	**1.89E-04**	**0.009742**	**3,945,412**	**3,951,090**
PotVar0117119	**1.89E-04**	**0.009742**	**5,370,258**	**5,161,430**
PotVar0089818	4.11E-04	0.018170	5,925,130	6,046,844
PotVar0125852	4.11E-04	0.018170	8,134,572	8,353,015
PotVar0085295	7.89E-04	0.029336	8,814,207	8,990,683
solcap_snp_c2_43535	7.89E-04	0.029336	9,273,361	9,439,555

#### Population 14 A-hwk

For all 54 phenotyped progeny clones of population 14 A-hwk, SNP genotyping data were obtained. The 815 SNP markers found to be heterozygous in the resistant parent CGN17888-16 (*S. hawkesianum)* were tested for association with resistance via a Kruskal‒Wallis test. Six SNP markers from 52.95 Mb to 59.13 Mb on chromosome 6 were significantly associated with PCN resistance, with FDR adjusted p values < 0.05. Two SNPs at 57.95 Mb and 58.22 Mb presented the lowest p values (*p* = 4.55E-05) (Table 6). The Pearson’s correlation coefficient was *r* = 0.53, indicating a strong effect of these SNPs on relative susceptibility. This strong effect is also visible in the box plot in Fig. 2B for the SNP PotVar0132843 (position 57948071). The distance between PotVar0132843 and the adjacent SNP markers was 4.15 Mb on the centromeric side and 0.9 Mb towards the telomere.

**Table 6:** SNP markers associated with resistance to *Globodera pallida* ‘Oberlangen’ in population 14 A-hwk. The markers define a resistance interval of 6.2 mb on chromosome 6. The SNP markers with the lowest p values are located at 57.95 and 58.22 mb in DM1-3 v4.3. The allele configuration of the SNP markers in population 14 A-hwk is shown in table S2

SNP	pVal	pValFDR	Position on Chr 06 DM1-3 v4.3	Position on Chr 06 DM1-3 v6.1
solcap_snp_c2_9002	6.46E-04	0.047838	52,947,838	53,030,113
solcap_snp_c2_9005	6.46E-04	0.047838	52,947,949	53,030,224
PotVar0040248	6.85E-05	0.010134	53,795,575	53,859,720
PotVar0132843	**1.23E-07**	**4.55E-05**	**57,948,071**	**57,639,347**
solcap_snp_c2_9220	**1.23E-07**	**4.55E-05**	**58,219,045**	**57,864,662**
solcap_snp_c1_15063	4.88E-07	1.20E-04	59,133,966	58,755,923

#### Population 15 A-spg

Sixty-nine clones of the 15 A-spg population were genotyped. Similar to the situation in 14 A-hwk and 5 A-gop, a single genomic region is significantly associated with PCN susceptibility in the 15 A-spg population. Analysis of 679 SNP markers heterozygous in the resistant parent CGN17602-2 (*S. spegazzinii)* revealed fourteen markers with FDR-adjusted p values below 0.05 in a genomic region between 46.64 Mb and 58.54 Mb on chromosome 6. The central SNP with the lowest p value of *p* = 2.064e-07 is PotVar0041167 at position 55,330,559 (bp). The neighbouring markers on either side are more than 2 Mb away (Table 7). Pearson’s correlation (*r* = 0.61) suggested a strong effect of PotVar0041167 (Fig. 2C).

**Table 7:** SNP markers significantly linked to *G. pallida* ‘Oberlangen’ resistance in population 15 A-spg are located within an interval of approximately 12 mb on chromosome 6. PotVar0041167 at 55.33 mb in DM1-3 v4.3 has the lowest p value. Table S3 shows the allele configuration of these markers in population 15 A-spg

SNP	pVal	pValFDR	Position on Chr 06 DM1-3 v4.3	Position on Chr 06 DM1-3 v6.1
PotVar0070000	4.22E-04	0.013231	46,641,588	46,904,074
solcap_snp_c1_15755	4.22E-04	0.013231	46,662,403	46,924,163
solcap_snp_c2_54191	4.22E-04	0.013231	46,662,915	46,876,107
solcap_snp_c2_5769	2.88E-04	0.013231	50,344,242	50,571,585
PotVar0040682	1.85E-06	1.32E-04	52,929,040	53,012,036
PotVar0040668	1.85E-06	1.32E-04	52,929,303	53,012,299
PotVar0040650	1.85E-06	1.32E-04	52,931,915	53,014,317
solcap_snp_c2_9001	1.85E-06	1.32E-04	52,947,389	53,029,664
solcap_snp_c2_9005	1.85E-06	1.32E-04	52,947,949	53,030,224
solcap_snp_c2_9009	1.85E-06	1.32E-04	52,951,567	53,033,012
PotVar0041167	**2.06E-07**	**1.03E-04**	**55,330,559**	**55,226,734**
solcap_snp_c2_9269	1.76E-04	0.010999	57,918,228	57,609,503
solcap_snp_c2_9220	3.52E-04	0.013231	58,219,045	57,864,662
solcap_snp_c2_9137	1.46E-03	0.035091	58,541,585	58,185,625

### Fine mapping of population 14 A-hwk by GBS analysis

The SNP array analysis revealed similar genomic locations for the resistance loci in populations 14 A-hwk and 15 A-spg between ~ 53 Mb and ~ 58 Mb. Owing to the low marker density around the central SNPs, it was not possible to determine whether the loci shared the same genomic position. To narrow the resistance region, the 14 A-hwk population had to be expanded. This analysis aimed to increase marker density and improve the resolution of the associated resistance loci.

#### Increasing the population

To expand the population 14 A-hwk and ensure reliable phenotyping, additional clones were preselected as follows: 200 clones were initially grown in small pots with peat soil in a greenhouse to assess plant vigour. Only vigorously growing clones were selected for resistance tests, leading to the eventual inclusion of 146 clones, each evaluated for resistance in at least two independent experiments. The relative susceptibility of these clones to the virulent *G. pallida* population ‘Oberlangen’ ranged from 0.1 to 74% (Figure S1). The results of the phenotyping experiments for population 14 A-hwk are listed in Supplementary Table S2.

#### GBS analysis in the extended 14 A-hwk population

GBS analysis was conducted on 146 14 A-hwk clones and parental clones. The total number of SNPs across all the samples was 58,936, with 19,784 SNPs fully covered in more than 66% of the samples with a minor allele frequency > 10%. Among these, 3,642 SNPs were found to be heterozygous in the *S. hawkesianum* parent, with 271 SNPs located on chromosome 6. Owing to deviating values in the Kosman genetic distance matrix (Kosman and Leonard 2005), progeny clones 14 A-hwk-193 and 14 A-hwk-199 were excluded from further analysis.

**Fig. 3:**
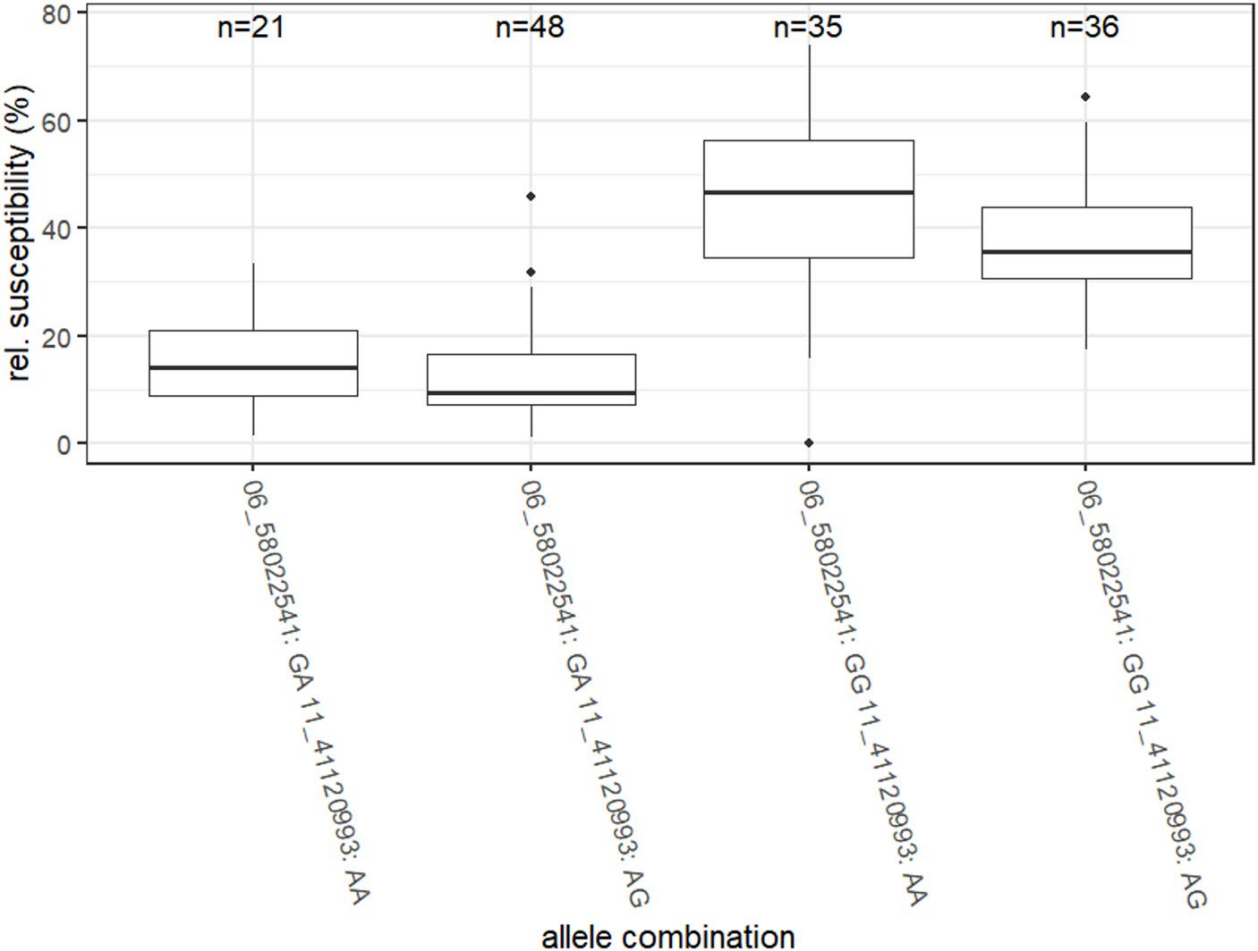
Boxplot of relative susceptibility to ’Oberlangen’ for all combinations of the GBS markers chr06_58022541 and chr11_41120993 based on marker alleles in 144 individuals of 14 A-hwk. The boxes represent the 25th and 75th percentiles, with medians indicated by bold lines

The analysis of the remaining 144 clones confirmed the location of the quantitative resistance locus (QRL) on chromosome 6, which was initially detected using the SNP array. Of the 172 SNPs linked to nematode resistance with p values (after FDR adjustment) < 0.05, 170 were located between 47.96 Mb and 58.92 Mb on LG06. The eighteen most significantly linked SNPs had p values < 1E-16, refining the QRL to a region between 56.5 Mb and 58.3 Mb (Table 8). Additionally, two SNPs with *p* < 0.05 (chr11*_*41120993 and Chr11_41121203, *p* = 0.02715542) were identified on LG11 at 41 Mb. Pearson’s correlation coefficient indicated a strong effect on resistance to *G. pallida* ‘Oberlangen‘ for the SNP Chr06_58022541 (r = 0.68) and a weak effect for the SNPs on chromosome 11 (r = 0.25). Compared with genotypes exhibiting homozygosity at these two loci, genotypes combining GA on chr06_58022541 and AG on chr11_41120993 presented a significantly lower relative susceptibility to *G. pallida* ’Oberlangen’ (Fig. 3**)**.

**Table 8:** GBS analysis of the 14 A-hwk population revealed a core resistance interval of 1.74 Mb on chromosome 6, with p values < 1E-16. The GBS markers with the lowest identical p value of 2.06E-17 are located between 57.43 and 58.02 Mb of DM1-3 v6.1

SNP	pVal	pValFDR
chr06_56534196	4.93E-19	9.58E-17
chr06_56820943	4.93E-19	9.58E-17
chr06_56952819	4.93E-19	9.58E-17
chr06_56979396	4.93E-19	9.58E-17
chr06_57215483	3.91E-19	9.58E-17
chr06_57433763	5.70E-20	2.06E-17
chr06_57433802	5.70E-20	2.06E-17
chr06_57433823	5.70E-20	2.06E-17
chr06_57433866	5.70E-20	2.06E-17
chr06_57519542	5.70E-20	2.06E-17
chr06_57555841	5.70E-20	2.06E-17
chr06_57559879	5.70E-20	2.06E-17
chr06_57560054	5.70E-20	2.06E-17
chr06_57988253	4.70E-20	2.06E-17
chr06_58022541	3.83E-20	2.06E-17
chr06_58022731	5.36E-19	9.68E-17
chr06_58266124	1.75E-19	5.27E-17
chr06_58266158	1.75E-19	5.27E-17

## Discussion

Pests and their host plants engage in an evolutionary ‘arms race’ to ensure survival and prosperity. As a result, new resistance develops in the plant, which in turn leads to new virulent pest populations that can overcome resistance, resulting in ‘boom and bust’ cycles (Brown and Tellier 2011).

Crop breeding therefore requires a constant search for new sources of resistance. In the past, potato breeders have often successfully used wild potato species and landraces to introduce major resistance genes to control important pests and diseases in potato cultivation. Examples include resistance to *Phytophthora infestans* from *S. demissum* (Bradshaw and Mackay 1994) and resistance to potato virus Y from *S. stoloniferum* and *S. tuberosum ssp. andigena* (Brigneti et al. 1997, Munoz et al. 1975).

Currently cultivated potato varieties that are resistant to *G. pallida* Pa2/3 contain resistance genes from only a few genetic sources, the most widespread being the QRL GpaV_vrn_ from *S. vernei*, which can be detected by the PCR marker HC (Rouppe van der Voort et al. 2000, Sattarzadeh et al. 2006; van Eck et al. 2017). Owing to the incomplete resistance effect of GpaV_vrn_, the associated resistance has already been overcome several times; virulent *G. pallida* populations have been detected in the field and in selection experiments after successive propagation cycles on a resistant potato genotype (Fournet et al. 2013; Mwangi et al. 2019b; Niere et al. 2014). Therefore, discovering new sources of resistance to the newly emerged ’Emsland’ virulence type is highly important for agricultural production. To our knowledge, this is the first report of resistance to this new virulence type of *G. pallida*. We characterised three out of the 17 accessions with resistance to *G. pallida* ‘Oberlangen’ via marker analyses in segregating populations. However, among the remaining 14 resistant accessions, some might carry resistance loci different from the loci we have reported here. Therefore, they constitute an invaluable resource for further analyses with the potential to broaden the genetic basis of resistance to new pathotypes of *G. pallida*.

Two different genomic locations were identified in the three characterised sources of resistance: one locus on the short arm of chromosome 5 and another on the long arm of chromosome 6. The resistance locus found in *S. gourlayi* ssp. *pachytrichum* GLKS 35354 is located in a known resistance ‘hotspot’ (Gebhardt and Valkonen 2001) on the short arm of chromosome 5. We therefore propose the designation GpaV_gop_. Many nematode resistance genes, including GpaV_vrn_, are located in this region. Some resistance genes, such as R1 and Rx2, which confer resistance to late blight and potato virus X, respectively, have been characterised and belong to the nucleotide-binding leucine-rich repeat (NLR) family of resistance genes (R genes) (Ballvora et al. 2002; Bendahmane et al. 2001). It is therefore possible that GpaV_gop_ is an NLR-type R gene.

QRLs of both *S. hawkesianum* CGN17888 and *S. spegazzinii* CGN17602 are located on the long arm of chromosome 6 and are therefore designated GpaVI_hwk_ and GpaVI_spg_, respectively. No resistance gene cluster is known here, although individual PCN resistance loci have been detected in this region. Caromel et al. (2003) described the weak QTL GpaM2 on chromosome 6 in an *S. spegazzinii* cross, whereas Gartner et al. (2024) reported a strong QRL against Pa2/3 at 57 Mb on chromosome 6 in *S. spegazzinii*. In both cases, the *S. spegazzinii* accessions differ from the CGN 17602 accession used in this study. Furthermore, the *G. pallida* populations used were of pathotype Pa2/3. However, it is yet to be determined whether this corresponds to the same genetic locus.

Despite the small population sizes for the *S. spegazzinii* population 15 A-spg and especially for the *S. gourlayi* ssp. *pachytrichum* population 5 A-gop, it was possible to define the resistance loci using SNPs, thus creating the prerequisite for producing diagnostic markers for use in marker-assisted selection. This will allow rapid use of these QRLs in the breeding process, which is urgently needed given the dire situation. Larger populations necessary to further narrow the location of the QRL are currently being generated by backcrossing.

A SNP array was employed to genotype the three segregating populations, offering several advantages over other genotyping methods. Notably, SNP arrays are cost-effective and require less effort than GBS for data evaluation while allowing robust allele calling across diverse populations (Darrier et al. 2019; Rasheed et al. 2017). Previous studies have successfully utilised SNP arrays originally designed for analysing commercial potato germplasms to investigate the genetic diversity of *Solanum* wild species panels (Duan et al. 2021; Hardigan et al. 2015). Our genotyping experiment effectively narrowed the resistance loci. However, the proportion of polymorphic SNPs in the *Solanum* wild species samples was significantly lower than that in the *S. tuberosum* samples. Consequently, fine mapping of the 14 A-hwk population was conducted via GBS.

In population 14 A-hwk, the expansion to over 140 individuals and their analysis by GBS confirmed the position of the major QRL on chromosome 6 from SNP analysis and closed marker gaps. As a result, a dense grid of sequences is now available for marker generation. In addition to the major QRL GpaVI_hwk_, the presence of an additional minor QRL on chromosome 11 at 41 Mb was revealed. The combination of the two genetic loci, as shown in Fig. 3, significantly enhances resistance by reducing the median relative susceptibility from 14.5 to 9%, suggesting that the minor quantitative resistance locus (QRL) is not an artefact. A similar locus was previously identified in the resistance mapping of *Solanum sparsipilum*, associated with the minor QRL GpaXI_spl_ (Caromel et al. 2005). Future studies should assess the effects of the combined loci GpaVI_hwk_ and GpaXI_hwk_ in further backcross progeny. It can be anticipated that incorporating these major and minor factors will significantly advance the development of resistant cultivars.

The identification of new resistance sources and the creation of diagnostic markers are critical steps in addressing the challenges posed by the newly emerged ’Emsland’ type virulent *G. pallida* populations. The findings of this study provide valuable insights and new opportunities for potato breeding, representing a significant advancement in the fight against nematode infestation. In particular, the new resistance loci identified here suggest new possibilities for the combination/pyramiding of nematode resistance loci and thus the creation of more durable resistance. The concept of pyramiding R genes is well established in resistance breeding (Mundt 2018; Pilet-Nayel et al. 2017). By stacking multiple resistance genes within a single cultivar, the genetic diversity and durability of resistance can be maximised. In particular, the locus on chromosome 5 identified in *S. gourlayi ssp. pachytrichum* and the loci on chromosome 6, derived from both *S. hawkesianum* and *S. spegazzinii*, present new opportunities to complement existing resistance to nematodes mediated, for example, by GpaV_vrn_.

However, to implement this strategy effectively, it is crucial to ascertain whether the loci from *S. hawkesianum* and *S. spegazzinii* represent distinct genes or allelic variations of a single gene. Determining the genetic relationship between these loci is essential for developing effective recombinants that combine both genes.

The successful stacking of newly identified loci or their combination with established resistance loci could provide dual benefits: enhancing immediate resistance against known virulent populations and extending the longevity of resistance by impeding the development of novel virulent nematode populations. This dual strategy is vital for sustainable crop protection, and further genetic analysis and breeding efforts are needed to realise its full potential. The emergence of virulent PCN populations illustrates that an evolutionary arms race within nematode populations can indeed occur when selection pressure is intense, rendering heavily used R genes ineffective. Only with co-protection through improved crop rotations will growers be able to benefit from the durability of novel resistance genes in future potato production.

## Supplementary Information

Below is the link to the electronic supplementary material.

Supplementary file1 (PDF 631 KB)

Supplementary file2 (XLSX 200 KB)

## Data Availability

The SNP datasets generated during this study are available in the figshare repository with the DOIs 10.6084/m9.figshare.28229168 (SNP array data) and 10.6084/m9.figshare.28239482 (GBS data for population 14 A-hwk). Raw sequencing reads of the GBS data have been deposited in the Sequence Read Archive (SRA) at NCBI (ncbi.nlm.nih.gov) under accession number PRJNA1260194.
