# Supplementary material for: Getting help from the extended family: identification and genetic characterisation of novel resistance to Globodera pallida ‘Oberlangen’ in wild Solanum species
Source: Mol Breed. 2025 Jul 22;45(8):63. doi: 10.1007/s11032-025-01582-0 (PMC12283489; doi:10.1007/s11032-025-01582-0)

Getting Help from the Extended Family: Identification and Genetic Characterisation of Novel Resistance to *Globodera pallida* 'Oberlangen' in Wild *Solanum* Species

Helgard Kaufmann[1], Sebastian Kiewnick[2], Thilo Hammann[3], Eckhard Tacke[4], Stefanie Hartje[4], Friedrich Kauder[5], Katja Muders[6], Vanessa Prigge[7], Marcus Linde[1] und Thomas Debener[1]

[1] Leibniz University Hannover, Institute of Plant Genetics, Section Molecular Plant Breeding, Hannover, Germany

helgard.kaufmann@genetik.uni-hannover.de

Figure S1: Frequency distribution of susceptibility to the *G. pallida* population 'Oberlangen' of the 14A-hwk population with 146 plants. The average of the third quartiles of the cyst counts from 2 to 3 experiments was used to calculate the susceptibility relative to the control 'Desiree'. Susceptibility values for the parental clones and the Pa2/3-resistant cultivar 'Cardoso' are indicated by black arrows.

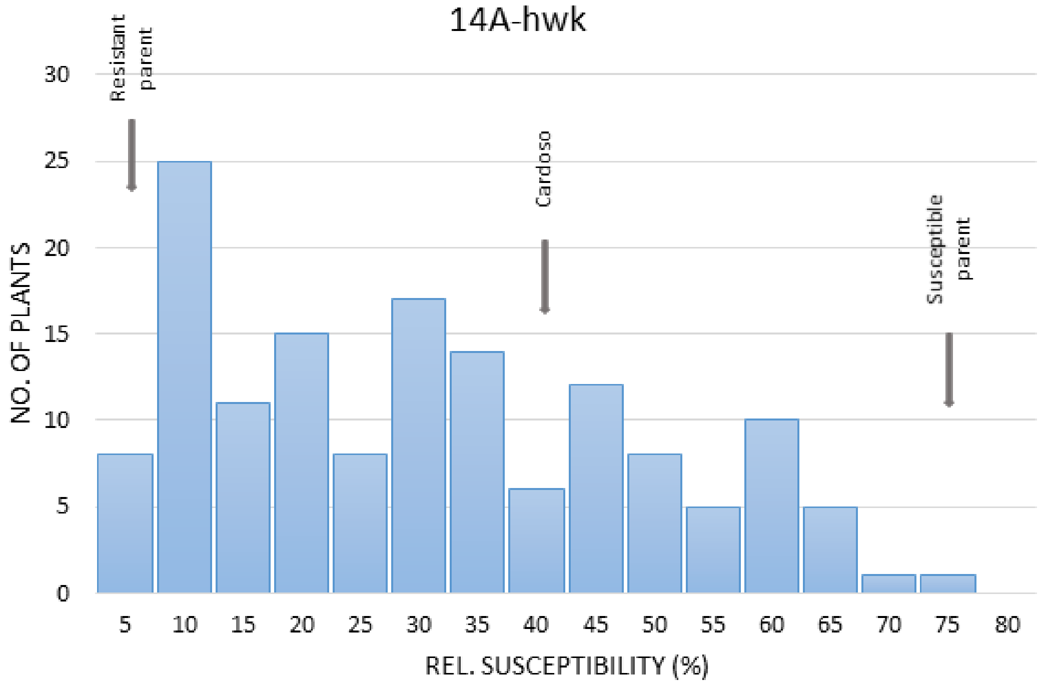

Supplement: Supplementary file 1 — Supplementary file1 (PDF 631 KB) [file 11032_2025_1582_MOESM1_ESM.pdf]
